# A biomechanical paradox in fish: swimming and suction feeding produce orthogonal strain gradients in the axial musculature

**DOI:** 10.1038/s41598-021-88828-x

**Published:** 2021-05-14

**Authors:** Yordano E. Jimenez, Richard L. Marsh, Elizabeth L. Brainerd

**Affiliations:** Department of Ecology and Evolutionary Biology, Brown University, 80 Waterman Street, Providence, RI 02912 USA

**Keywords:** Animal physiology, Biomechanics, Ichthyology

## Abstract

The axial musculature of fishes has historically been characterized as the powerhouse for explosive swimming behaviors. However, recent studies show that some fish also use their ‘swimming’ muscles to generate over 90% of the power for suction feeding. Can the axial musculature achieve high power output for these two mechanically distinct behaviors? Muscle power output is enhanced when all of the fibers within a muscle shorten at optimal velocity. Yet, axial locomotion produces a mediolateral gradient of muscle strain that should force some fibers to shorten too slowly and others too fast. This mechanical problem prompted research into the gearing of fish axial muscle and led to the discovery of helical fiber orientations that homogenize fiber velocities during swimming, but does such a strain gradient also exist and pose a problem for suction feeding? We measured muscle strain in bluegill sunfish, *Lepomis macrochirus,* and found that suction feeding produces a gradient of longitudinal strain that, unlike the mediolateral gradient for locomotion, occurs along the dorsoventral axis. A dorsoventral strain gradient within a muscle with fiber architecture shown to counteract a mediolateral gradient suggests that bluegill sunfish should not be able to generate high power outputs from the axial muscle during suction feeding—yet prior work shows that they do, up to 438 W kg^−1^. Solving this biomechanical paradox may be critical to understanding how many fishes have co-opted ‘swimming’ muscles into a suction feeding powerhouse.

## Introduction

**Figure 1:**
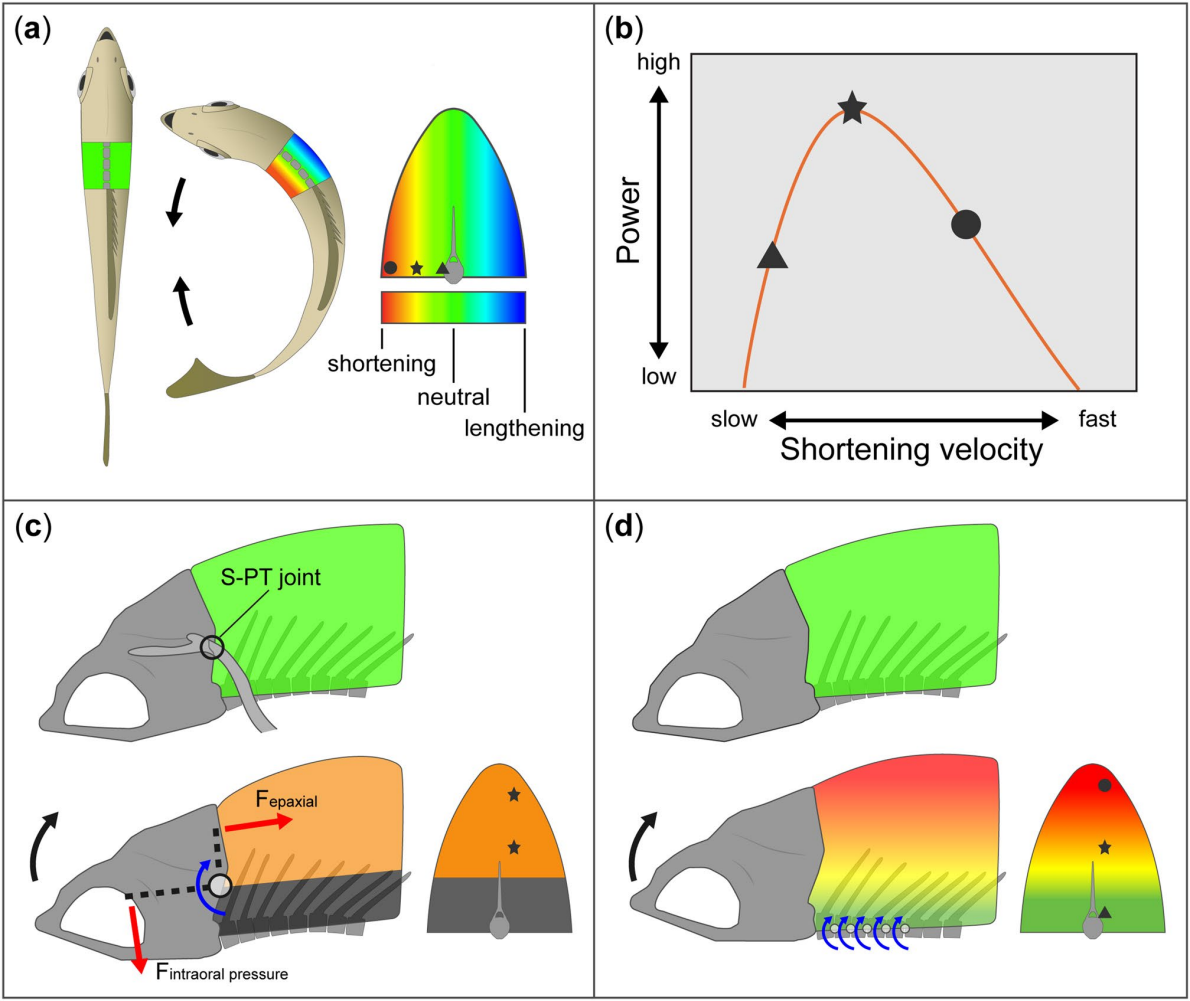
Patterns of longitudinal strain in the epaxial musculature for swimming and suction feeding. (**a**) Axial locomotion: lateral body flexion produces a mediolateral gradient of longitudinal strain in the axial muscle mass. The neutral axis, the vertebral column, undergoes neither shortening nor lengthening. If the muscle fibers were oriented longitudinally, they would also experience this strain gradient. (**b**) A diagrammatic power-velocity curve, illustrating how a gradient of fiber strain rate would affect power production. Muscle shortening velocities in some regions would be faster (circle) or slower (triangle) than those needed for generating maximal power (V_opt_), and only some regions (star) would shorten at V_opt._ (**c**, **d**) Alternative models of epaxial mechanics in suction feeding. (**c**) The lever model of neurocranial elevation for suction feeding^30^. Epaxial forces acting on the in-lever generate neurocranial elevation, intraoral forces acting on the out-lever resist neurocranial elevation, and the fulcrum occurs at the level of the S-PT joint in the pectoral girdle. Epaxial muscle dorsal to this joint shortens uniformly to produce neurocranial elevation, and therefore could potentially all contract at V_opt_, but muscle ventral to this joint (dark grey) cannot contribute to cranial elevation. (**d**) An alternative, the beam model of neurocranial elevation for suction feeding, where neurocranial elevation is produced by the dorsiflexion of multiple intervertebral joints. All muscle dorsal to the vertebral column can shorten to produce neurocranial elevation, but the amount of longitudinal shortening is largest in the dorsal region and approaches zero at vertebral column. We test this hypothesis of a dorsoventral gradient in suction feeding in this study. Abbreviation: S-PT, supracleithral and post-temporal bones.

Whether an animal is swimming, jumping, running or flying, the mechanical output of muscle is one of the key drivers of performance. Hence, studying the conditions in which muscle contractions produce maximal force, work, and power is critical to understanding how animals execute the behaviors that enable them to survive^1–5^. When chasing prey or evading predators, fish accelerate through the water using their axial musculature to flex the body from side to side^6–9^. As the axial muscles contract and bend the body, lateral body flexion produces a mediolateral gradient of strain within the axial muscle mass, with the greatest shortening occurring near the skin and the least shortening near the backbone^10–13^ (Fig. 1a). Beam-like bending of an axial musculature with longitudinal muscle fiber orientations (i.e., the null morphology) would produce heterogeneous fiber strains and velocities, and given the power-velocity properties of muscle^14,15^, only a thin band of fibers would shorten at velocities that generate high powers (Fig. 1b). As a result, this gradient is thought to severely limit muscle power output. The detrimental mechanical implications of this null morphology prompted investigations into the mechanical role of the helical fiber orientations observed in fish and shark axial muscle. Multiple studies have shown that these complex muscle fiber orientations form a sophisticated gearing system that makes muscle fiber strain and shortening velocity more homogeneous than predicted by beam-like bending of the null morphology^10,11,16–19^. This strain-homogenizing fiber architecture is thought to enable high power outputs from fibers throughout the whole muscle mass, independent of their distance from the vertebral column (i.e., the neutral axis). This hypothesis is supported by numerous studies showing that fish generate very high power from the white axial musculature during fast-starts, rapid evasive and predatory swimming maneuvers^6–8,20^.

In addition to locomotion, many fishes have co-opted the axial musculature for suction feeding^21–24^. Species like largemouth bass (*Micropterus salmoides*) and bluegill sunfish (*Lepomis macrochirus*) generate over 90% of their suction power by actively shortening most of the ‘locomotor’ muscle mass^23–26^. Suction feeding studies have typically modelled epaxial mechanics with the assumption that the epaxial muscle mass functions like muscle belly actuating a lever system (Fig. 1c)^27–32^. This lever model has yielded valuable insights for comparative studies of suction feeding performance across species^29–32^. The lever model infers from specimen manipulation that the fulcrum is at the level of a joint within the pectoral girdle and makes the simplifying assumption that the input muscle force acts on a single point of the neurocranium, implying that epaxial muscle shortens uniformly^30^. The lever model is challenged by the finding that neurocranial elevation usually involves dorsal flexion of multiple intervertebral joints, suggesting smooth bending rather than hinge-like rotation occurs^33^. Hence, we hypothesize that suction feeding is powered by beam-like bending that produces a dorsoventral gradient of longitudinal strain in the epaxial musculature, where muscle strain decreases from dorsal to ventral during any given bout of muscle shortening (Fig. 1d).

A dorsoventral gradient of longitudinal strain in the epaxial muscle mass (hereafter also referred to as muscle strain) during feeding would be anatomically orthogonal to the mediolateral gradient in swimming. If muscle fiber architecture is indeed specialized to homogenize fiber strain within the musculature as it undergoes heterogeneous longitudinal strain during locomotion, high muscle power outputs are unlikely during suction feeding because the muscle fibers would be oriented to counteract a mediolateral gradient, not a dorsoventral gradient. Conversely, if the architecture were specialized for equalizing fiber strain along a dorsoventral gradient, this would likely impede locomotor power production. Thus, behaviors with motions that produce anatomically orthogonal strain gradients in the muscle might have gearing solutions that conflict with each other, preventing the muscle from generating maximum power output for both locomotion and suction feeding, and perhaps limiting peak muscle performance to only one of these vital behaviors. Here, we measured muscle shortening using sonomicrometry to determine whether the dorsal half of the axial musculature, the epaxial musculature, of bluegill sunfish exhibits a dorsoventral gradient of longitudinal strain in suction feeding and a mediolateral gradient of longitudinal strain in locomotion.

## Results

**Figure 2:**
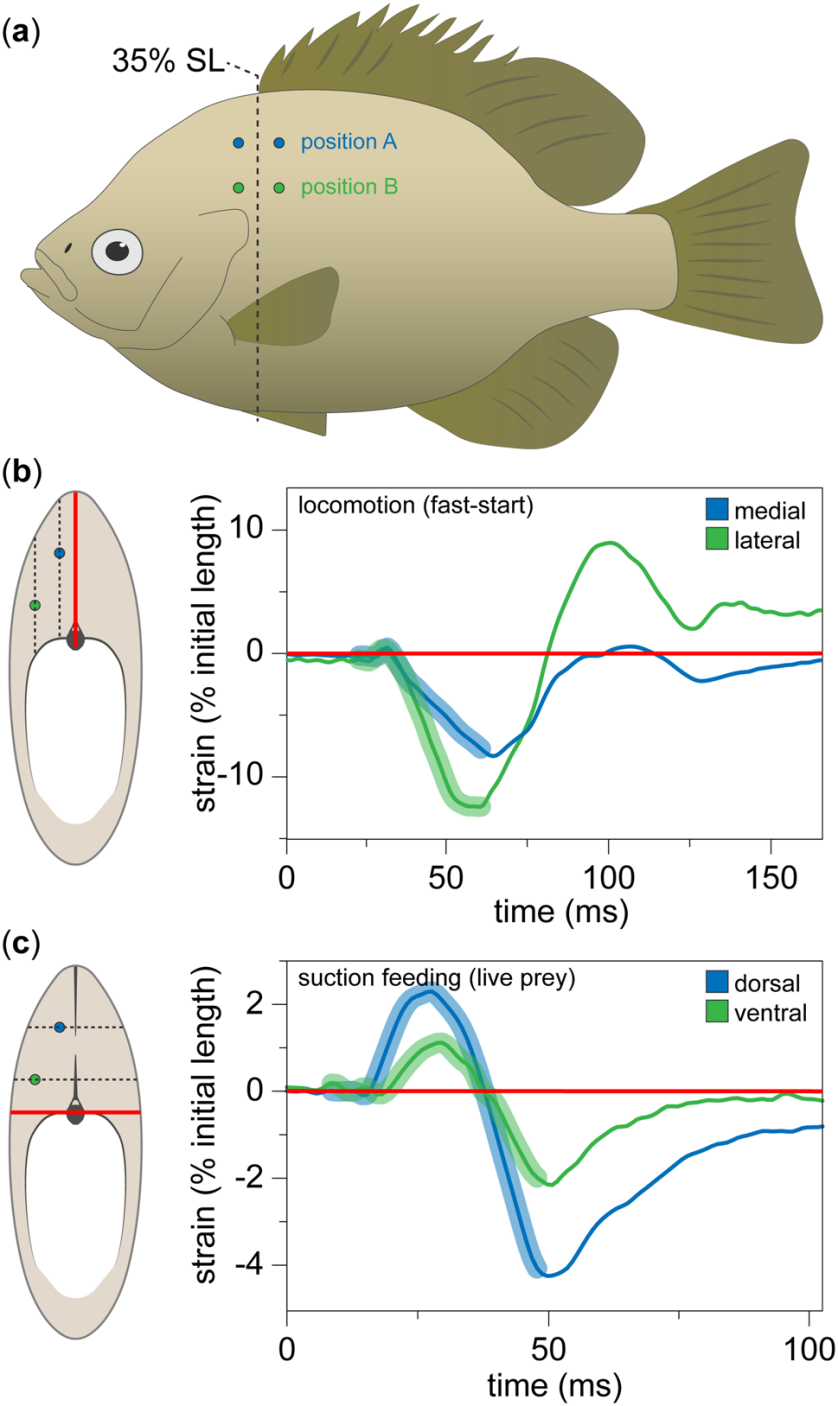
Sonomicrometer implantation sites and *in vivo* muscle length dynamics. (**a**) Lateral view of the implantation sites for each sonomicrometer pair. (**b**) Epaxial length dynamics during an evasive fast-start alongside a transverse view of sonomicrometer positions relative to the neutral axis of bending (solid red line). In lateral flexion, longitudinal strain is expected to vary as a function of mediolateral distance from the neutral axis, and thus the dorsoventral position of the instrument can be ignored. (**c**) Epaxial length dynamics during a suction feeding on live prey alongside a transverse view of sonomicrometer positions. In dorsiflexion, longitudinal strain is expected to vary as a function of dorsoventral distance from the neutral axis (solid red line), and thus the mediolateral position of the instrument can be ignored. Lengthening before shortening, as shown here, occurred in some but not most feeding strikes. Thick lines indicate the duration of muscle activity (see supplementary Fig. S1 for sample EMG traces). Data shown are from individual Lm01.

**Figure 3:**
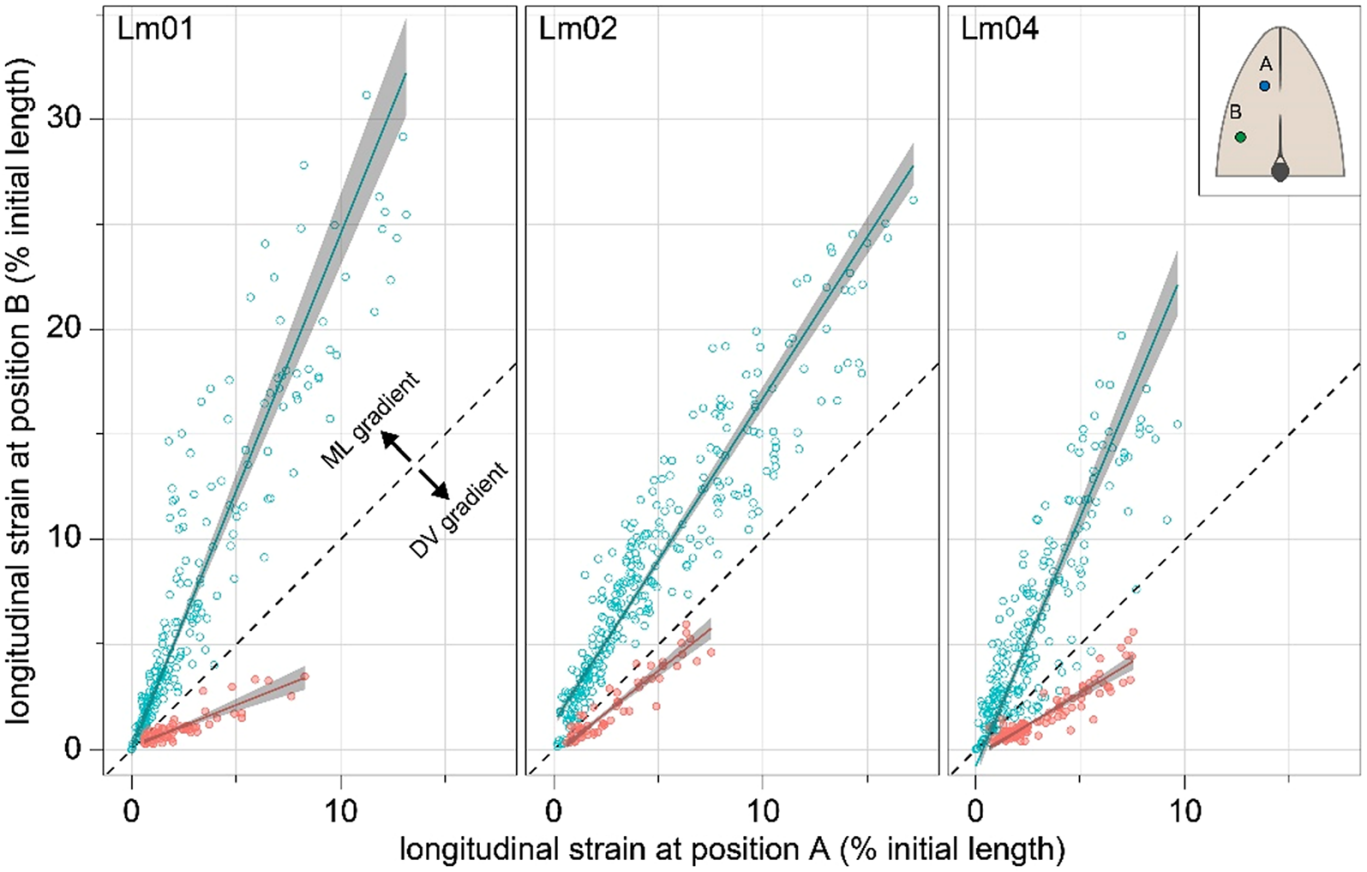
Feeding produces a strain gradient anatomically orthogonal to swimming. Longitudinal strain measured at the ventrolateral location (‘**B**’) as a function of strain measured at the dorsomedial location (‘**A**’) for three individual *L. macrochirus* (Lm01, 02 and 04). Locomotion: open blue circles. Feeding: closed red circles. In the absence of either mediolateral or dorsoventral strain gradients, the strains at the two locations are predicted to be equal as is indicated by the dashed lines with a slope of 1.0. Regression lines are shown with 95% confidence intervals. Regression coefficients, equations, and statistics for linear regressions can be found in Table 1. Inset shows positions of (‘**A**’) and (‘**B**’) relative to the neutral axes of feeding and locomotion (orthogonal black lines). *ML* mediolateral, *DV* dorsoventral.

**Figure 4:**
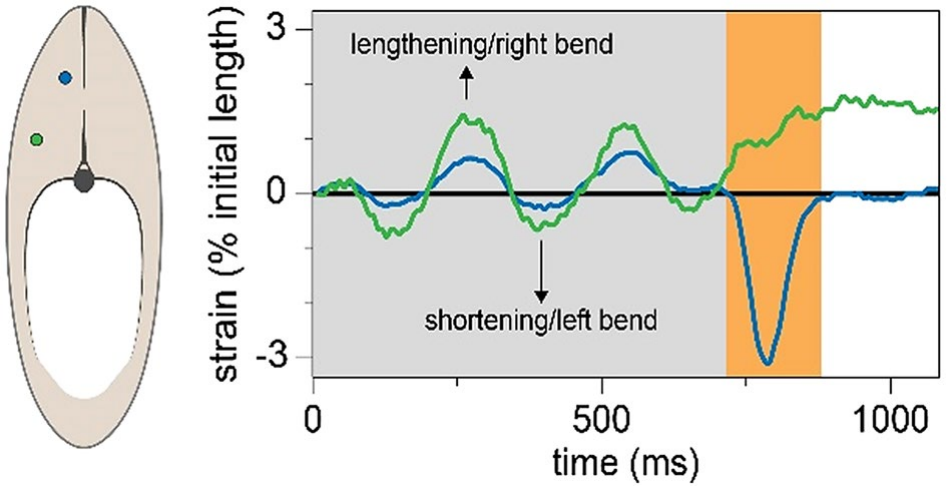
Muscle length dynamics in burst swimming followed by mixed swimming and feeding behavior. Plot shows a sprint (grey area) followed by a strike with body bending to the right (orange area). The sprint sequence shows the expected cycles of muscle lengthening and shortening, and more importantly, the higher magnitudes of longitudinal strain the lateral sonomicrometer (green) relative to the medial sonomicrometer pair. The sprint sequence is then followed by a strike on pellet food with simultaneous lateral flexion to the right, which shows the more complex yet expected pattern of high muscle shortening in the dorsal region (blue) with muscle lengthening in the lateral region (green). Data shown are from individual Lm01.

We found that locomotor behaviors produced a mediolateral gradient of longitudinal strain in the epaxial muscle, while feeding behaviors produced a dorsoventral gradient of longitudinal strain (Figs. 2 and 3; see supplementary Fig. S1 for sample EMG traces). If longitudinal muscle strain were homogeneous, strain would be the same at the dorsoventral position (‘A’) and mediolateral position (‘B’), independent of their different distances from the neutral axes of bending. In this case, the slopes of the regressions in Fig. 3 would be equal to one. However, linear regressions showed that all three individuals had slopes statistically significantly greater than one during locomotion, indicating that longitudinal strain was larger in the lateral muscle region. Conversely, linear regressions showed that all three individuals had slopes statistically significantly less than one during feeding, indicating that longitudinal strain was larger in the dorsal muscle region (Fig. 3 and Table 1; significance based on the 95% confidence intervals not overlapping with a slope of one). Finally, although we excluded non-planar feeding behaviors from our formal analysis, we found that side strikes (feeding behaviors with simultaneous dorsiflexion and lateral flexion) produced muscle length dynamics consistent with the finding of anatomically orthogonal strain gradients (Fig. 4).

**Table 1:** Summary of major axis regression analysis.

Individual	N	r^2^	Slope	y-intercept	*P*-value (two tailed)	Confidence interval (2.5% intercept)	Confidence interval (97.5% intercept)	Confidence interval (2.5% Slope)	Confidence interval (97.5% Slope)
**Swimming**									
Lm01	222	0.86	2.45	0.08	< 0.0001	− 0.34	0.46	2.32	2.59
Lm02	299	0.88	1.54	1.29	< 0.0001	0.98	1.59	1.48	1.61
Lm04	231	0.76	2.38	− 0.82	< 0.0001	− 1.31	− 0.38	2.21	2.56
**Feeding**									
Lm01	63	0.76	0.39	0.14	< 0.0001	0.01	0.27	0.34	0.45
Lm02	55	0.88	0.80	− 0.25	< 0.0001	− 0.50	− 0.01	0.72	0.89
Lm04	108	0.84	0.60	− 0.34	< 0.0001	− 0.50	− 0.19	0.55	0.65

## Discussion

### The Paradox

Our discovery of a dorsoventral strain gradient in feeding presents a paradox: bluegill sunfish should not be able to attain high power outputs from their epaxial muscle in suction feeding, but they do. Bluegill sunfish can generate exceptionally high epaxial muscle power, up to 438 W kg^−1^, in the most powerful strikes^26^. What makes this a paradox? Within the current paradigm, muscle fiber architecture is specialized to equalize fiber strain mediolaterally for axial locomotion. Thus, this same architecture is not expected to be able to equalize fiber strain dorsoventrally in feeding. Yet, the seeming contradiction (i.e., the paradox) is that bluegill sunfish do generate very high axial muscle power despite the expectation that a dorsoventral gradient should prevent them from doing so.

The current paradigm of fish axial locomotion and white muscle mechanics has argued and shown to varying degrees that (1) lateral body flexion causes the whole musculature to deform heterogeneously along a mediolateral gradient, (2) muscle fiber orientations in the axial musculature are specialized to allow for homogeneous fiber strain within a whole muscle undergoing heterogeneous strain during locomotion because (3) heterogeneous fiber strain is mechanically detrimental. We regard point one as uncontroversial, since it has been demonstrated repeatedly across different species, including here in bluegill. We regard points two and three as assumptions that, if rejected or refined, can resolve the paradox in bluegill sunfish and provide a new biomechanical insight. Point two could be rejected if epaxial fiber gearing in bluegill sunfish were specialized for suction feeding, not swimming. Point two could also be rejected if epaxial fiber gearing were specialized for both swimming and suction feeding with a sophisticated, albeit unknown, gearing system that can homogenize strain for anatomically orthogonal gradients. Finally, point three could be rejected if bluegill sunfish were capable of generating peak power for both behaviors without any specialized fiber gearing. Here we consider each of these solutions to the paradox briefly.

The first possibility is that, in bluegill sunfish, gearing of the epaxial muscles is specialized for power production during feeding and cannot produce high muscle power output during swimming. Very high muscle power is routinely observed in fishes during fast-starts^6–8,34^, but fast-start power has not been measured in bluegill sunfish. Bluegill sunfish may have responded to selective pressures favoring a muscle fiber architecture that homogenizes fiber strain along the dorsoventral axis to maximize muscle power output for prey capture. This scenario would challenge the prevailing and well-supported view that most fish maximize axial muscle power output during fast-starts^6–8^, and future studies would be needed to determine whether bluegill sunfish actually do achieve high mass-specific powers during fast-starts. The second possibility is that bluegill sunfish do indeed maximize muscle power for both behaviors, but require a sophisticated gearing system that can homogenize fiber strain for orthogonal gradients. Since gearing is determined by fiber angulation and three-dimensional muscle deformation^35–37^, the former of which cannot change in a given organism, the only possible way to produce orthogonal gearing gradients would be if the bulging or shearing within the muscle mass was different for each behavior.

Both of the aforementioned solutions assume that having a gradient of fiber strain (i.e., fiber strain heterogeneity) is as problematic as previously suggested^11,19^. The problem of heterogeneous fiber strain has often relied on assessing muscle performance based on experimentally derived isotonic muscle properties, yet some studies have shown the limitations of this approach. Even operating under this assumption, power-velocity curves can have a broad plateau where muscle fibers not shortening at velocities that generate peak power (V_opt_) can still generate high power^3^. In addition to isotonic properties, V_opt_ can vary based on the frequency, strain trajectory, and activation patterns of the behavior, among many other factors^3,38^. Due to these complex interactions, the sensitivity of *in vivo* power production to fiber strain heterogeneity is unclear.

### Implications for suction feeding muscle mechanics

Most suction feeding studies have assumed or implied uniform muscle strain when modelling epaxial muscle mechanics (Fig. 1c)^29,30,32^. Contrary to this assumption, we show that suction feeding produces a dorsoventral gradient of longitudinal strain in the epaxial musculature of bluegill sunfish (Fig. 1d), where muscle strain varies along the dorsoventral axis of the cross-section of muscle (Figs. 2 and 3). Yet much of our current understanding of epaxial mechanics is based on muscle strain measurements that we now know do not represent the entire muscle. If strain had been measured in a ventral epaxial region, longitudinal muscle strain would be lower in magnitude and muscle function would have been interpreted differently, as active shortening would imply positive power production (i.e., a motor), but active contractions with minimal shortening would imply isometric force production (i.e., a strut or stabilizer). Of course, all of this assumes a null morphology of longitudinal muscle fibers, which is certainly not the case in the axial muscle of fishes^11,18,19^. Hence, conclusions about *in vivo* epaxial mechanics should be made cautiously when using longitudinal strain. Determining *in vivo* muscle mechanics in suction feeding may require quantifying the variables that have been shown to influence the gearing of muscle—fiber orientations and *in vivo* muscle deformation^35,36^.

### Side strikes: mixed swimming and feeding behaviors

Bluegill sunfish occasionally bent their heads to the side while attacking live prey that were not located directly in front of the mouth. These side strikes were characterized by dorsiflexion with simultaneous lateral flexion (Fig. 4). Although we excluded such feeding trials from our formal analysis in order to analyze lateral and dorsal bending separately, they illustrate the complexity of muscle length dynamics during behaviors involving simultaneous motion in two planes. These muscle length dynamics are both implied by the orthogonal gradients in planar feeding and planar swimming (Fig. 3) and evident in otherwise anomalous longitudinal strain data (Fig. 4). Suction strikes with simultaneous dorsal and lateral flexion are distinguished from sequences of behaviors with relatively discrete motions, such as lateral flexion for acceleration followed by dorsiflexion for suction feeding followed by lateral flexion for deceleration^39^. Among many interesting points, how exactly do side strikes affect power production? Does the concave side of the body (where muscle shortens) contribute positive power to cranial expansion? Conversely, does the convex side generate negative power, thereby resisting cranial expansion? While these questions are difficult to answer, comparing performance of planar strikes and side strikes may be a feasible way of doing so. Considering the complexity of the physical environments that many fish species inhabit, side strikes may be, along with planar strikes, an important part of the food capture repertoire of fishes in the wild.

### Methodological considerations

We suspect that longitudinal muscle strain for suction feeding is a linear function of distance from the neutral axis, although we could not determine the linearity of this relationship with the spatial distribution of our sampling. Determining linearity would require measuring longitudinal strain in at least three positions at different distances from the neutral axis of interest and ideally at the same distances from the neutral axis of the other behavior—such that all strain recordings are equally affected by non-planar motions. For example, a robust configuration for detecting a linear strain gradient in suction feeding would involve measuring longitudinal strain at a dorsal, middle, and ventral position, with each instrument positioned close to the vertical septum where longitudinal muscle strain is low for swimming. A related issue is whether the measurements of midline curvature in conjunction with beam theory can be used to calculate longitudinal muscle strain. This concept has been successfully implemented in locomotion studies but requires a dorsal or ventral view of fish swimming in order to calculate midline curvature from a digitized outline of the body^12,40–42^. Of critical importance to this technique is the observation that the midline, calculated using the left and right edges of the body, is an accurate estimate of the vertebral column’s position. Such methodology is not feasible for dorsiflexion during feeding as the dorsal and ventral edges of the body are not equidistant from the vertebral column, and so a calculated midline curvature would not accurately estimate vertebral curvature. Although it is not feasible to estimate longitudinal strain with a lateral view of feeding, techniques such as fluoroscopy, rotoscoping, and XROMM (X-ray Reconstruction of Moving Morphology), could be used to test the relationship between vertebral flexion and longitudinal epaxial strain. Even so, this relationship may not be simple because the epaxial muscles are physically connected with the hypaxial muscles, the ventral half of the axial musculature that plays a complementary but distinct kinematic role in suction feeding—actuation of the hyo-pectoral interface^43^. Finally, quantitatively associating the neutral axis of axial dorsiflexion with a discrete anatomical structure is complicated by the kinematic variability of suction feeding behaviors at both the individual and species levels, although the available evidence suggests that the neutral axis of bending is approximately at the level of the vertebral column^33^.

### Concluding remarks

Past studies have only examined the problem of heterogeneous fiber strain in the axial musculature of fishes within the context of locomotion. Our findings suggest that we must broaden our investigation of strain heterogeneity to include suction feeding. Strain heterogeneity may be particularly problematic for species that require high mass-specific power outputs from the axial musculature, such as bluegill sunfish^26^. In contrast, strain heterogeneity may be less problematic for species that activate only a small cross-sectional area of the epaxial muscle during suction feeding (largemouth bass)^24^, and those that generate relatively low mass-specific power from axial muscle during suction feeding (largemouth bass and channel catfish)^26,44^. Interspecific variation of swimming and suction feeding performance is well-known in fishes, but here we show how the muscle mechanics for high-performance swimming and high-performance suction feeding may be at odds. Indeed, orthogonal strain gradients may create mechanical constraints and tradeoffs between axial locomotion and suction feeding in fishes. Investigating the dual functionality of this muscle will elucidate how many fishes have successfully co-opted, and perhaps in some cases mechanically specialized, the ‘locomotor’ muscle for generating powerful feeding behaviors that are crucial for survival.

## Methods

### Animals and training protocol

Bluegill sunfish (*Lepomis macrochirus*) were caught at Morses Pond in Wellesley, Massachusetts. Fish (standard length 173, 171, and 161 mm for Lm01, Lm02, and Lm04, respectively) were housed at Brown University in tanks at room temperature. We acclimated the fish for a minimum of six weeks, during which time they were trained to feed on carnivore pellets and live prey, such as goldfish (*Carassius auratus*) and rosies (*Pimephales promelas*). All procedures were approved by the Institutional Animal Care and Use Committee (IACUC) of Brown University and followed guidelines and policies set forth by the IACUC of Brown University. Reporting of methods and results followed ARRIVE guidelines^45^.

### Sonomicrometer positions

We anesthetized and CT scanned each individual *in vivo* to measure the mediolateral and dorsoventral distances from the vertebral column at the intended implantation sites. Sonomicrometers were positioned to measure strain at different distances from the neutral axes of bending, not to measure strain within a single myomere. Each sonomicrometer transducer was mounted on a custom-made stainless steel holder with three arms^46^ that allowed the transducer to be positioned at the desired muscle depth. We implanted two pairs of transducers (1 mm diameter) in the epaxial musculature on the left side of the body at approximately  35% standard length (SL; Fig. 2a). Each pair was approximately 15 mm apart and parallel to the long-axis of the animal, defined as a line going from the snout tip to the notch in the caudal fin. One pair was implanted dorsomedially at approximately 14 mm dorsal to the vertebral column and 3 mm lateral to midline. The other pair was implanted ventrolaterally at approximately 8 mm dorsal to the vertebral column and 5 mm lateral to the midline. After each experiment, we anesthetized and CT-scanned each individual to confirm sonomicrometer positions.

### Surgical procedures

Fish were anesthetized via immersion in 0.12 g/L buffered MS-222 (Tricaine methanesulfonate). We then placed the fish in a surgical tray with a flow of anesthetic solution, and intubated the mouth to flow oxygenated water over the gills. For each sonomicrometer (4 total per individual), we removed scales from the region of implantation, made a small dermal incision (1 mm), and used a 16-gauge needle with a blunted tip to make a path for the sonomicrometer-holder unit. We then sutured the external arms of the holder onto the skin. As a part of a related project, we also implanted three electrodes in the epaxial muscle on each side of the body at approximately 35% SL. The electrode and sonomicrometers wires exiting from the muscle were glued together (E600 flexible craft adhesive) to form a common cable, which we then sutured onto the region above the head to prevent the sonomicrometers from dislodging.

### Data collection

Muscle length (L) data were recorded at a sampling rate of 1041 Hz in SonoLab software (version 3.4.81) using a Sonometrics system (Model TR-USB Series 8). We measured water temperature to get the muscle temperature in our ectothermic fish, and input the appropriate speed of sound at the beginning of each experiment to account for any temperature changes^47^. We synchronized strain recordings and light video by using a LabChart PowerLab (Model PL3516) to send a 1 Hz signal to the sonomicrometry acquisition software and a flashing LED light in the field of view of the cameras. We elicited various swimming (turns [i.e., non-fast start body bends], sprints and fast-starts) and feeding behaviors (suction feeding on live prey and pellets, coughs, and chews) that require varying degrees of axial flexion. We used synchronized recordings to classify behaviors and to exclude trials in which the fish moved in more than one plane (e.g., suction feeding strikes with concomitant lateral flexion). Post-processing of the sonomicrometer signals was done in Igor Pro (Wavemetrics) and recordings were smoothed using the smooth.spline function in the stats package in R^48^.

### Statistical analysis

Muscle strain was calculated as (L-Li)/Li, where L is muscle length and Li is initial muscle length, the muscle length prior to the onset of the behavior and muscle shortening or lengthening. Each trial consists of a full, half, or quarter wavelength of lengthening or shortening, depending on the behavior. Each data point is the absolute value of peak strain measured at both positions during the behavior. As neither the dorsomedial nor ventrolateral position is an independent variable, we performed a model 2 major axis linear regression using the *lmodel2* package in R^49.^ A major axis regression is appropriate for our data because both the dorsomedial and ventrolateral positions are expected to have similar measurement errors, and the relationship between X and Y is symmetric^50^. Data from each individual were analyzed separately, since variation in sonomicrometer implantation was expected to impact the slope of the regressions. Regression results and confidence intervals can be found in Table 1.

## Supplementary Information

Supplementary Information.

## Data Availability

Strain data for Fig. 3 are available in Dryad. Sonomicrometry and video data used in this study are available on the Zoological Motion Analysis Portal upon request (zmaportal.org, Study Identifier ZMA27).
