# Supplementary material for: A biomechanical paradox in fish: swimming and suction feeding produce orthogonal strain gradients in the axial musculature
Source: Sci Rep. 2021 May 14;11:10334. doi: 10.1038/s41598-021-88828-x (PMC8121803; doi:10.1038/s41598-021-88828-x)

**Supplementary Material**

**A biomechanical paradox in fish: swimming and suction feeding produce orthogonal strain gradients in the axial musculature**

Yordano E. Jimenez^1*^, Richard L. Marsh^1^, and Elizabeth L. Brainerd^1^

^1^Department of Ecology and Evolutionary Biology, Brown University, 80 Waterman Street, Providence, RI 02912, USA

*Correspondence to: Email: jimenez.yordano@gmail.com (Y.E.J.)

**Figure S1.** Sample EMG traces showing epaxial muscle activity in locomotion and feeding. Timing of muscle length changes are indicated by red arrows. Data were sampled at 4000 Hz and EMG signals were filtered with a bandwidth of 100 – 1000 Hz. Traces correspond to data shown in Fig. 2


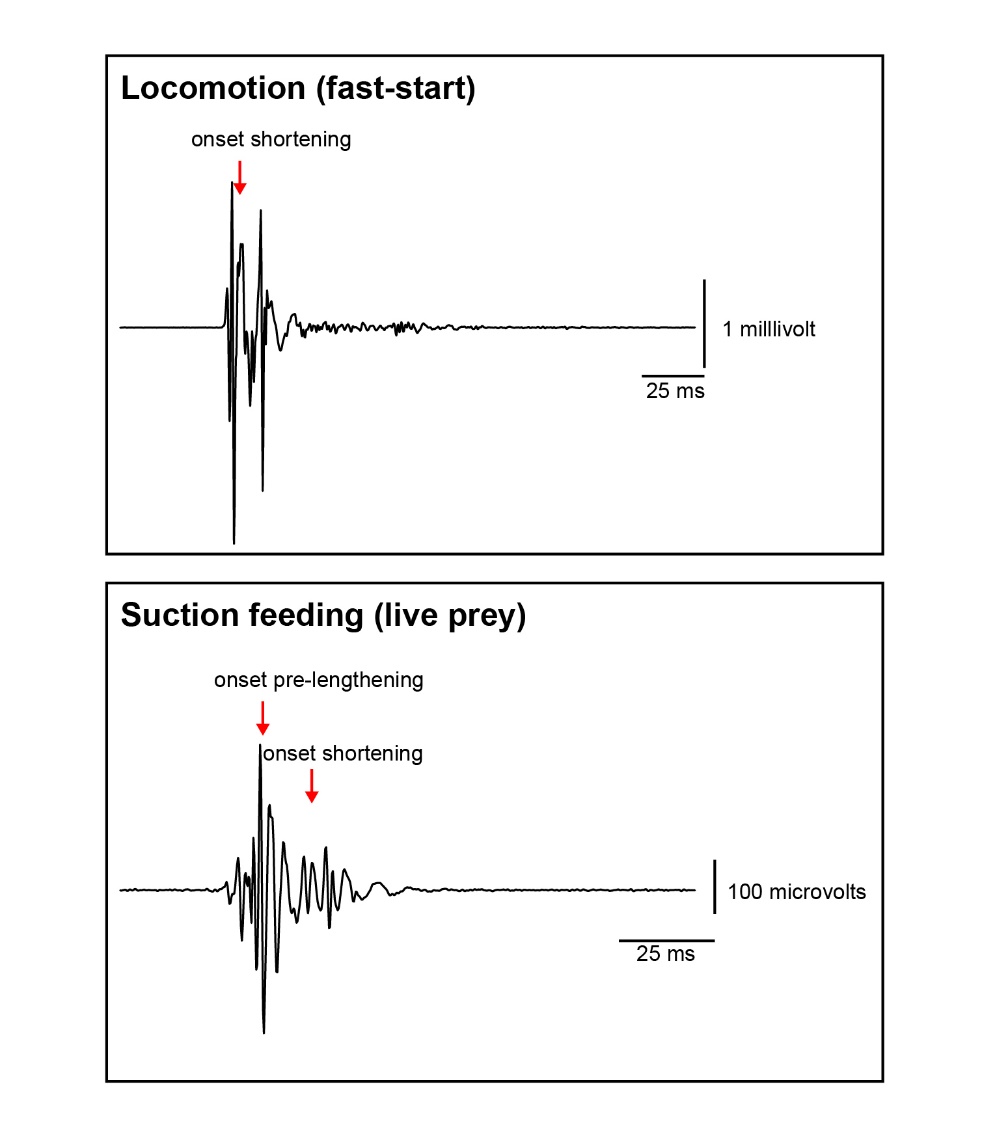

Supplement: Supplementary file 1 — Supplementary Information. [file 41598_2021_88828_MOESM1_ESM.docx]
